# Regulatory Non-coding RNAs for Death Associated Protein Kinase Family

**DOI:** 10.3389/fmolb.2021.649100

**Published:** 2021-08-06

**Authors:** Qingshui Wang, Youyu Lin, Wenting Zhong, Yu Jiang, Yao Lin

**Affiliations:** ^1^ Central Laboratory at the Second Affiliated Hospital of Fujian Traditional Chinese Medical University, Collaborative Innovation Center for Rehabilitation Technology, Fujian University of Traditional Chinese Medicine, Fuzhou, China; ^2^ Key Laboratory of Optoelectronic Science and Technology for Medicine of Ministry of Education, College of Life Sciences, Fujian Normal University, Fuzhou, China; ^3^ Prenatal Diagnosis Centre, Women and Children’s Hospital, School of Medicine, Xiamen University, Xiamen, China

**Keywords:** DAPK, non-coding RNA, miRNA, lncRNA, circRNA

## Abstract

The death associated protein kinases (DAPKs) are a family of calcium dependent serine/threonine kinases initially identified in the regulation of apoptosis. Previous studies showed that DAPK family members, including DAPK1, DAPK2 and DAPK3 play a crucial regulatory role in malignant tumor development, in terms of cell apoptosis, proliferation, invasion and metastasis. Accumulating evidence has demonstrated that non-coding RNAs, including microRNA (miRNA), long non-coding RNA (lncRNA) and circRNA, are involved in the regulation of gene expression and tumorigenesis. Recent studies indicated that non-coding RNAs participate in the regulation of DAPKs. In this review, we summarized the current knowledge of non-coding RNAs, as well as the potential miRNAs, lncRNAs and circRNAs, that are involved in the regulation of DAPKs.

## Death-Associated Protein Kinase Family

**FIGURE 1:**
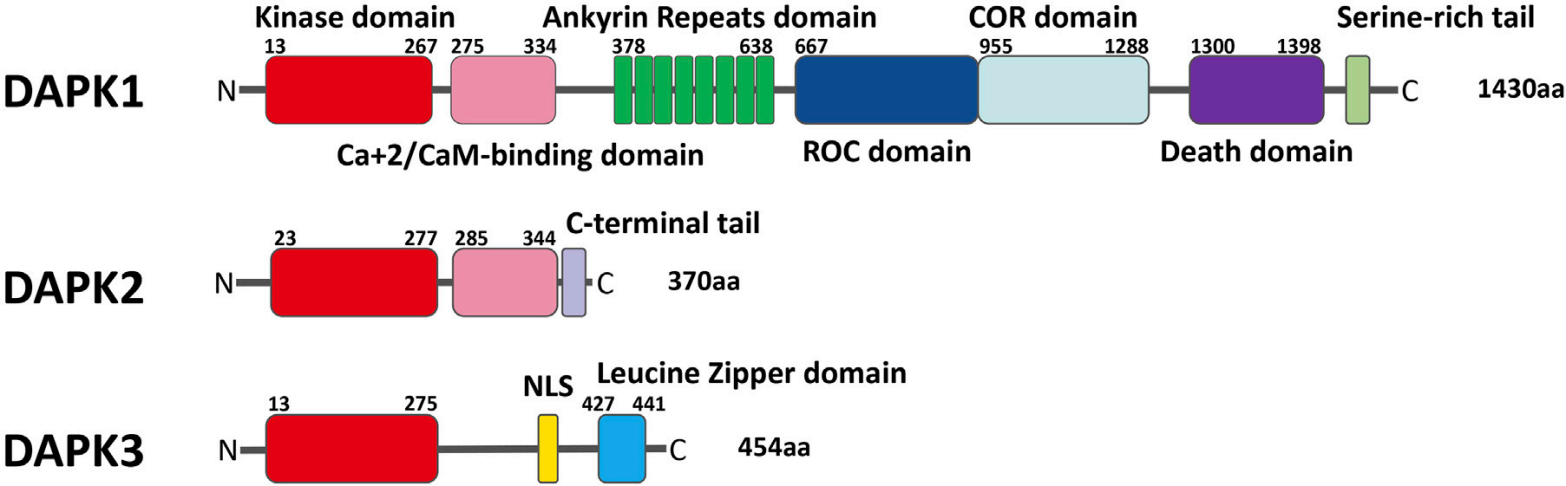
Schematic representation of the domains of each of the DAPK family members with their respective location.

DAPK is a family of serine/threonine kinases that belong to the calmodulin regulated kinase super family (Huang et al., 2014). At present, the most studied DAPK family member is DAPK1 (Lin et al., 2009; Stevens et al., 2009; Chen et al., 2014; Fujita and Yamashita, 2014; Yung et al., 2019). DAPK1 was identified in an unbiased antisense based genetic screen for genes whose protein products were necessary for interferon gamma (IFN-γ) induced death in HeLa cells (Lai and Chen, 2014; Liu et al., 2016). DAPK1 has a unique multi-domain structure, and the sequence from the N end to the C end is: a kinase domain, a calmodulin regulatory domain, eight continuous ankyrin repeats, two potential P-loop binding regions of Ras of Complex proteins (C-terminal of ROC), a death domain and a serine-rich tail. DAPK2 is comprised of a kinase domain, a calmodulin-binding autoregulatory domain and a C-terminal tail. DAPK3 contains kinase domain, a leucine zipper domain, and two putative nuclear localization sequences (NLS) (Shiloh et al., 2014) (Figure 1).

DAPK1 is involved in the regulation of cell apoptosis, autophagy and migration (Lin et al., 2007; Harrison et al., 2008; Gandesiri et al., 2012; Rennier and Ji, 2013a; Rennier and Ji, 2013b; Benderska et al., 2014; Rennier and Ji, 2015; Tian et al., 2016). Previous research revealed that DAPK1 is a tumor suppressor gene, and the level of DAPK1 was down regulated in various cancers (Raval et al., 2007; Kilinc et al., 2012; Gao et al., 2015; Cai et al., 2017; Xie et al., 2017; Steinmann et al., 2019). In the early stage of cancer, DAPK1 can suppress tumor growth and metastasis by increasing apoptosis. In late cancer, DAPK1 can inhibit cell movement and adhesion by interfering with integrins (Kuo et al., 2006). In contrast to DAPK1, DAPK2 has not been identified as a tumor suppressor in solid tumors. DAPK2 is mainly expressed in the hematopoietic compartment. At present, DAPK2 has been found to act as a tumor suppressor in several types of leukemia (Rizzi et al., 2007; Humbert et al., 2014; Ye et al., 2016). DAPK3 is considered as a tumor suppressor. Previous reports revealed that the DAPK3 promoter is often methylated in various types of cancer, resulting in the loss of its tumor suppressor effect (Brognard et al., 2011; Das et al., 2016).

## Methylation of Death-Associated Protein Kinase Gene in Tumor

Hypermethylation of DAPK has been frequently described in many different cancers compared with normal tissues, including esophageal, breast malignancies, head and neck, kidney and bladder, ovary, B cell lymphoma and lung cancer (Sanchez-Cespedes et al., 2000; Lehmann et al., 2002; Brock et al., 2003; Christoph et al., 2006; Collins et al., 2006). Raveh et al. found that the methylation of DAPK1 gene accounts for about 20% in primary colon cancer patients. Bing et al. found that the methylation frequency of DAPK1 gene was approximately 17.7 and 54.8% in normal gastric tissue and gastric cancer tissue samples respectively (Ye et al., 2012). Harder et al. revealed that the methylation of DAPK1 gene accounted for about 68% in 34 liver cancer samples, but in 16 normal liver tissues, its methylation accounted for about 31% (Harder et al., 2008). In addition, Flatley et al. revealed that cervical cancer cells whose DAPK1 gene is methylated may be more susceptible to human papillomavirus (HPV) infection (Flatley et al., 2014).

Abnormal promoter methylation is an early and frequent event in the process of cell carcinogenesis, and can be used as a sensitive biomarker of tumorigenesis. Bing et al. pointed out that the methylation frequency of the DAPK1 promoter in gastric cancer tissue samples was three times higher than that in normal gastric tissue. That is, along with the process of gastric carcinogenesis, the methylation rate of DAPK1 gene will increase, so it can be speculated that DAPK1 can be used as a marker of gastric cancer (Ye et al., 2012). Research by Krajnovic et al. pointed out that DAPK1 gene methylation accounted for 79%, and O-6 methylguanine DNA methyltransferase (MGMT) gene methylation accounted for 59% in 32 follicular lymphoma (FL) samples. And the synergistic methylation of these two genes can be used as a marker of FL disease recurrence and drug resistance (Krajnović et al., 2013).

Although there have many relevant reports on the detection of the methylation frequency of the DAPK1 promoter in multiple patient samples. However, due to the limited patient information and the number of samples. There are few studies on the analysis of the methylation of DAPK1 gene, the mRNA level of DAPK1, and the protein level of DAPK1 in the same patient sample. It is necessary to simultaneously detect these three levels in appropriate samples to further understand the biological function and clinical significance of DAPK1 gene methylation in tumors. Our previous study found that DNA methylation status of DAPK1 did not correlate well with its mRNA or protein expression in breast cancer, which indicated that DAPK1 expression is not only regulated by methylation (Zhu et al., 2017). In addition to the methylation, there has been an increasing number of studies on the association between regulation of DAPKs and non-coding RNAs.

## Non-coding RNAs

In recent years, non-coding RNA (ncRNA) has received increasing attention (Guo et al., 2015; Esposito et al., 2016; Sun et al., 2018; Fridrichova and Zmetakova, 2019; Ban et al., 2020; Zhang et al., 2020). With more and more non-coding RNAs being identified, their functions have been gradually discovered. In 1993, Ambros and Ruvkun announced the discovery of miRNA which is related to the development and regulation of Caenorhabditis elegans. After that, hundreds of similar short RNAs were found in *drosophila*, human and worm cells (Cech and Steitz, 2014). Non-coding RNAs regulate target genes at transcription level and RNA processing level, and participate in almost all physiological processes such as embryo development, protein synthesis, cell differentiation and apoptosis (Cech and Steitz, 2014). There are two major types of non-coding RNA with regulatory functions according to their size: short chain non-coding RNAs (siRNA, miRNA and piRNA) with length between several and 200 nt, and long chain non-coding RNAs (lncRNA) with length greater than 200 nt. In recent years, a number of non-coding circular RNAs (circRNAs) have also been identified (Hsiao et al., 2017). Currently, miRNA, lncRNA and circRNA are the most studied non-coding RNAs.

**FIGURE 2:**
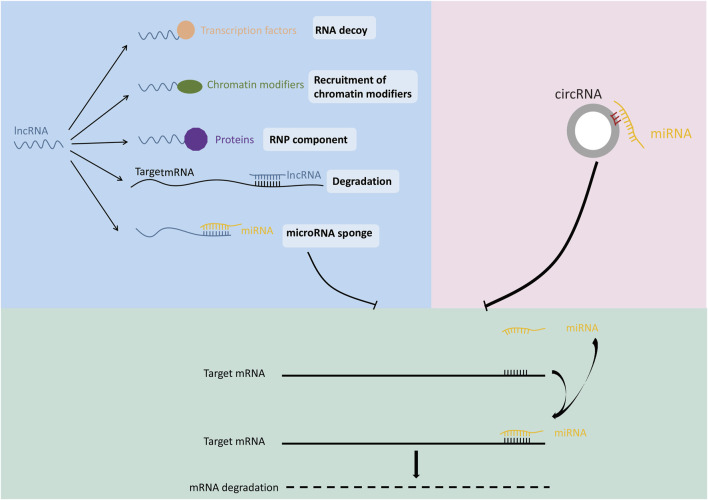
Mechanisms of ncRNA function.

MiRNA is a family of highly conserved, non-coding RNAs encoding 19–25 nt. The main function of miRNA is its participation in the regulation of gene expression after transcription (Wang et al., 2018a). MiRNA can play a suppressive role by binding to the mRNA of target gene sequence often locating on the 3′UTR, thereby inhibiting target gene expression (Figure 2) (Bushati and Cohen, 2007; Chen et al., 2019; Michlewski and Cáceres, 2019; Sur et al., 2019). Dysregulation of miRNA has been confirmed to be closely related to cancer and many other diseases (Liu and Li, 2019; Zhao et al., 2019). Previous studies revealed that the expression patterns were different for each miRNA among different cancer tissues. For example, miR-34 was down-regulated in prostate cancer, breast cancer, lung cancer and osteosarcoma compared with normal tissues, but was up-regulated in liver cancer (Zhang et al., 2019a). These studies suggested that same gene may have a completely different expression pattern in different types of tumors due to the regulation of miRNA.

LncRNA refers to non-coding RNA transcripts with length greater than 200 nt (Jathar et al., 2017). With the continuous progress of genomics and exploration of lncRNA family members, the regulatory effect of lncRNA on gene function has been gradually discovered (Chen, 2016). LncRNAs exert functions at different positions according to different mechanisms such as regulation of transcription, translation, protein modification and the formation of RNA-protein or protein-protein complexes (Figure 2) (Xing et al., 2015; Botti et al., 2017; Renganathan and Felley-Bosco, 2017; Wang et al., 2018b; Liao et al., 2018). The competing endogenous RNA (ceRNA) is the most studied mechanism of lncRNA-mediated gene expression regulation. LncRNA can act as a miRNA sponge *via* ceRNA mechanism, thereby regulating the expression of miRNA target genes (Iwakiri et al., 2017; Militello et al., 2017; Chen et al., 2018a). For example, lncRNA ATB was deregulated in hepatocellular carcinoma, lung cancer and other malignant tumors, and caused tumor occurrence and development through ceRNA mechanism (Li et al., 2017a). LncRNA DANCR was deregulated in lung cancer, and promoted tumor growth and invasion by binding with tumor suppressor miR-216a (Zhen et al., 2018).

CircRNA is a new type of non-coding RNA with covalently closed ring structure. The formation of circRNA is through the reverse splicing process in the splicing modification of precursor mRNA, which makes the splicing donor at the 3’end of pre-mRNA connect with the splicing receptor at the 5′ end (Chen and Yang, 2015). Since circRNA involves a wide range of biological processes, the deregulation of circRNA may lead to abnormal cell function and diseases (Zhang et al., 2018a; Bi et al., 2018; Chen et al., 2018b; Lin and Chen, 2018; Zhang et al., 2019b). For example, the expression of circSLC8A1 was decreased in bladder cancer. Overexpression of circSLC8A1 inhibited bladder cancer cell proliferation, migration and invasion. Further study revealed that circSLC8A1 acts as a miRNA sponge for miR-494 and miR-130b, and subsequently regulate the expression of their target gene PTEN (gene of phosphate and tension homology deleted on chromsome ten), thereby suppressed the progression of bladder cancer (Lu et al., 2019). Similarly, a large amount of circRNAs have been identified to enhance the transcription of target genes through ceRNA mechanisms (Figure 2) (Zhang et al., 2018b; Fang et al., 2018; Zhu et al., 2018; Cao et al., 2019; Costa et al., 2019; Liu et al., 2019; Yu and Liu, 2019; Yuan et al., 2019; Chen et al., 2020).

## The Regulatory Non-coding RNAs for Death-Associated Protein kinases

### Non-coding RNAs Involved in the Transcription Regulation of Death-Associated Protein kinase1

Emerging evidence showed that numerous miRNAs caused DAPK1 deregulation in multiple cancers, for example, miR-103, miR-34a-5p and miR-191 etc.

In colorectal cancer, Chen et al. found that the increased levels of miR-103 and miR-107 were related to the metastasis potential of tumor cells by targeting DAPK1 and KLF4 (Kruppel like factor 4). In renal cell carcinoma (RCC), the expression of DAPK1 was decreased. Functionally, DAPK1 overexpression repressed RCC cell proliferation, migration and invasion. Meanwhile, the expression of DAPK1 was identified to be regulated by miR-34a (Jing et al., 2019). In ovarian cancer, knockdown of DAPK1 could weaken its response to TNF-αinduced cell death in CRL-7566 cells. Higher level of miR-191 in ovarian cancer patient samples compared with controls was verified, and miR-191 was confirmed to directly target DAPK1 and regulate its expression using luciferase assay. Further, the authors demonstrated that the miR-191-DAPK1 axis plays a major role in modulating the response of endometrioid carcinoma cells to death-inducers (Tian et al., 2015; Vastrad et al., 2017). In gliomas and glioblastoma, miR-103a-3p-DAPK1 axis and miR-22-3p-DAPK1 axis might be associated with the diagnosis and treatment of gliomas and glioblastoma (Vastrad et al., 2017). In nasopharyngeal carcinoma (NPC), the over-expression of miR-483-5p decreased the radiosensitivity of NPC cells *in vivo* and *in vitro*. MiR-483-5p decreased radiation-induced apoptosis and DNA damage, and increased NPC cell colony formation by targeting DAPK1 (Tian et al., 2019). These results implied that modulation of miR-483-5p-DAPK1 axis may provide a new approach for increasing the radiosensitivity of NPC. In pancreatic cancer, the level of DAPK1 was significantly down-regulated, and increased DAPK1 expression inhibited the migration and invasion of pancreatic cancer cells. MiR-182 was highly expressed in pancreatic cancer and confirmed to directly target DAPK1. MiR-182-DAPK1 axis has been demonstrated to plays an important role in the development and progression of pancreatic cancer (Xu et al., 2017). In endometrial cancer (EC), lncRNA MIR22 was reported to be significantly down-regulated in endometrial cancer tissues. Functionally, over-expression of lncRNA MIR22 significantly inhibited endometrial cancer cells proliferation, induced EC cells apoptosis, and arrested endometrial cancer cells in G0/G1 phase. Furthermore, miR-141-3p was identified as a target for lncRNA MIR22. Subsequently, lncRNA MIR22 was found to inhibit endometrial cancer cell proliferation by regulating the miR-141-3p-DAPK1 axis (Cui et al., 2018). In breast cancer, hypermethylation of miR-127 was observed in 58 breast cancer tissues compared with paired normal breast tissues. Clinical research data indicated that the up-regulated expression levels of DAPK1 in breast cancer patients were negatively correlated with the decreased expression of miR-127-5p. In addition, the miR-127-DAPK1 axis was found to be related to breast cancer progression, particularly metastasis (Pronina et al., 2017).

**FIGURE 3:**
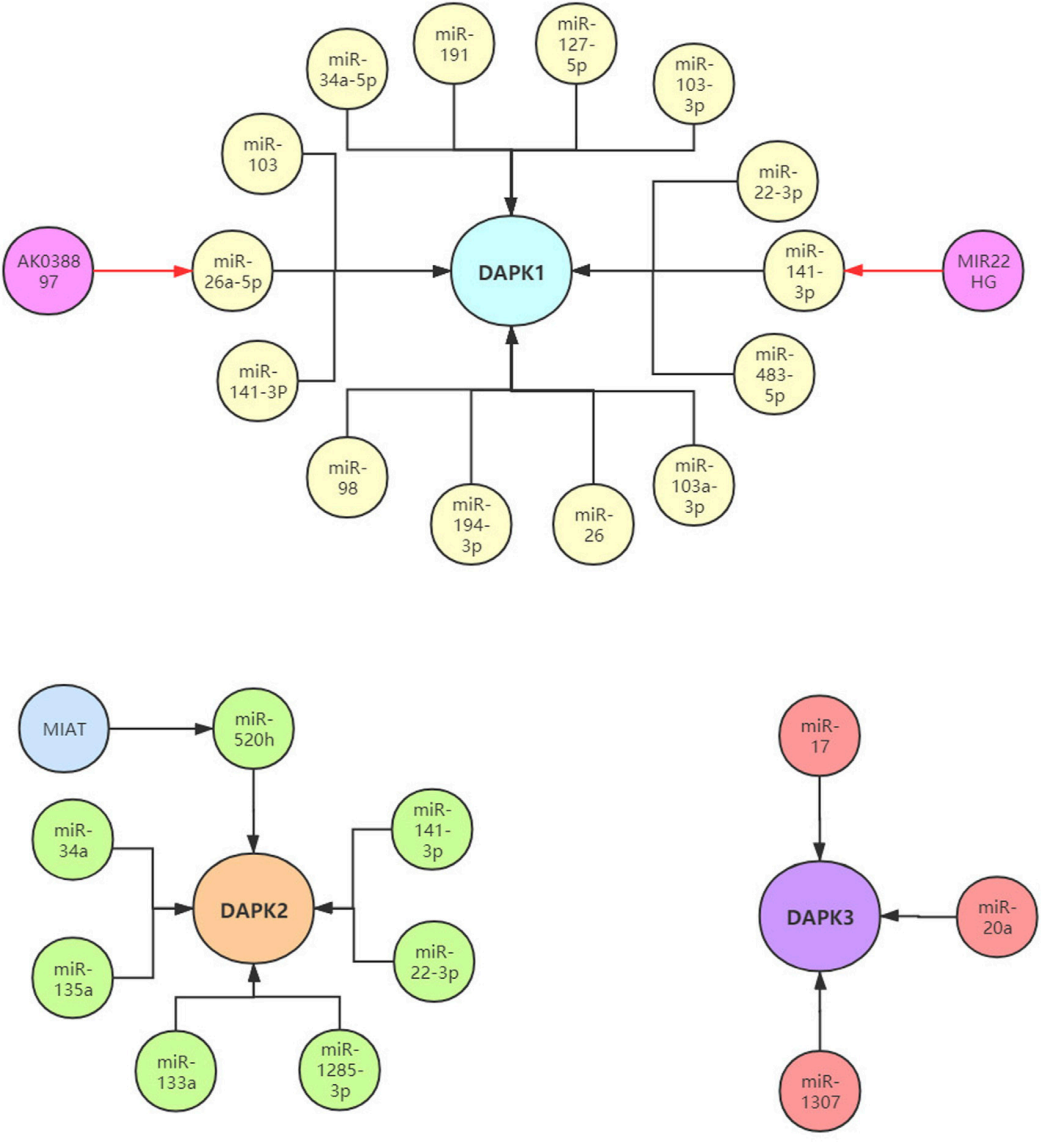
The reported non-coding RNAs in the regulation of DAPKs.

In addition to cancer, the expression of DAPK1 was also regulated by non-coding RNAs in numerous other diseases. In rheumatoid arthritis (RA), miR-103a-3p was significantly up-regulated in the whole blood and peripheral blood mononuclear cells of RA patients compared with healthy control (Anaparti et al., 2017). Additionally, DAPK1 was found to be a target of miR-103a-3pand decreased in RA. In Parkinson’s disease (PD), the level of DAPK1 was increased in PD mice and positively correlated with synucleinopathy, and DAPK1 is a target of miR-26. MiR-26 knockdown or over-expression of DAPK1 resulted in synucleinopathy, dopaminergic neuron cell death, and motor disabilities in wild-type mice. Further investigation revealed that miR-26-DAPK1 axis were essential in the formation of the molecular and cellular pathologies in PD (Su et al., 2019). In cardiac ischemia-reperfusion (IR) injury, the level of miR-98 was decreased in the cardiomyocytes subjected to hypoxia/reoxygenation (H/R) and in the myocardium of the I/R rats. Moreover, increased miR-98 expression could significantly reduce the myocardial oxidative stress, ischemic injury and cell apoptosis. Subsequently, DAPK1was confirmed as a direct target of miR-98 using luciferase activity assay (Zhai et al., 2019). In chronic obstructive pulmonary disease (COPD), PM2.5 significantly aggravated apoptosis in cigarette-inflamed Human bronchial epithelial cells (HBEpiCs). The level of miR-194-3p was detected to be down-regulated in PM2.5-CSS-treated HBEpiCs. Bioinformatics and luciferase activity assay reported that DAPK1 was directly targeted by miR-194-3p. Inhibition of miR-194-3p increased DAPK1 expression and apoptosis in normal HBEpiCs (Zhou et al., 2018). In ischemic stroke, increased lncRNA AK038897 and decreased miR-26a-5p levels were observed in mouse brains following middle cerebral artery occlusion/reperfusion (MCAO/R) and in neuro-2A (N2a) neuroblastoma cells following oxygen-glucose deprivation and reoxygenation (OGD/R). Further studies showed that AK038897 and DAPK1 were targets of miR-26a-5pusing luciferase activity assay. Finally, AK038897 -miR-26a-5p- DAPK1 axis was considered as a key mechanism controlling cerebral ischemia injury (Wei et al., 2019). In polycystic ovary syndrome (PCOS), the level of miR-141-3p was decreased in the ovaries of rat PCOS models, and increased level of miR-141-3p decreased cells apoptotic rate (Li et al., 2017b). Luciferase activity assay reported that DAPK1 was the target of miR-141-3p, indicating that miR-141-3p is involved in the etiology of PCOS by targeting DAPK1 (Table 1; Figure 3).

**TABLE 1:** Non-coding RNAsin the regulation of DAPKs.

Number	Cancer or Disease	Target gene	Non-coding RNAs
1	Colorectal cancer	DAPK1	miR-103
2	Renal cell carcinoma	DAPK1	miR-34a-5p
3	Ovarian cancer	DAPK1	miR-191
4	Breast cancer	DAPK1	miR-127-5p
5	Gliomas and glioblastoma	DAPK1	miR-103a-3p、miR-22-3p
6	Nasopharyngeal carcinoma	DAPK1	miR-483-5p
7	Endometrial cancer	DAPK1	MIR22HG/miR-141-3p
8	Rheumatoid arthritis	DAPK1	miR-103a-3p
9	Parkinson’s disease	DAPK1	miR-26
10	Cardiac ischemia-reperfusion	DAPK1	miR-98
11	Chronic obstructive pulmonary disease	DAPK1	miR-194-3p
12	Ischemic stroke	DAPK1	AK038897/miR-26a-5p
13	Polycystic ovary syndrome	DAPK1	miR-141-3p
14	Epithelial ovarian cancer	DAPK2	miR-520g
15	Breast cancer	DAPK2	miR-520h
16	Gastric cancer	DAPK2	miR-34a、miR-135a
17	Colorectal cancer	DAPK2	miR-1285-3p
18	Diabetic cardiomyopathy	DAPK2	MIAT/miR-22-3p
19	Ischemia reperfusion injury	DAPK2	miR-133a
20	Breast cancer	DAPK3	miR-17/20a
21	Ovarian cancer	DAPK3	miR-1307

### Non-coding RNAs Involved in the Transcription Regulation of Death-Associated Protein kinase2

The expression of DAPK2 was also reported to be regulated by non-coding RNAs in cancers and other diseases. In epithelial ovarian cancer (EOC), the level of miR-520g was significantly increased in EOC tissues compared with normal tissues, and over-expression of miR-520g increased EOC cell proliferation and promoted cell invasion (Zhang et al., 2016). DAPK2 was a target of miR-520g. MiR-520g knockdown or DAPK2 over-expression reduced EOC cell proliferation, invasion and chemoresistance. These results suggested that miR-520g promoted EOC progression and drug resistance by regulating DAPK2. In breast cancer, miR-520h could promote the drug resistance of breast cancer cells (Su et al., 2016). Bioinformatics prediction, compensatory mutation and functional validation further confirmed that DAPK2 is a target of miR-520h. Over-expression of DAPK2 could abolish breast cancer cell drug resistance induced by miR-520h, suggesting miR-520h-DAPK2 axis is a potential functional target for breast cancer therapy. In gastric cancer, the level of miR-34a was significantly increased and correlated with dendritic cell mediated enhancement of anti-tumor immunity against gastric cancer cell. DAPK2 and Sp1 were targets of miR-34a (Yan et al., 2016a). Another research found that miR-135a was highly expressed in gastric cancer tissues compared to normal tissues. Gastric cancer with high miR-135a level was more likely to display aggressive characteristics. Moreover, miR-135a promoted the proliferation and invasion of oxaliplatin-resistant gastric cancer cells, and inhibited E2F transcription factor 1 (E2F1) induced apoptosis by inhibiting E2F1 and DAPK2 expression (Yan et al., 2016b). In colorectal cancer, miR-1285-3p improved colorectal cancer cell proliferation and escape from apoptosis by targeting DAPK2 (Villanova et al., 2020). In diabetic cardiomyopathy (DCM), lncRNA myocardial infarction associated transcript (MIAT) was significantly upregulated in the rat model of DCM. Knockdown of MIAT reduced cardiomyocyte apoptosis and improved left ventricular function in diabetic rats. Luciferase activity assay reported that MIAT and DAPK2 were directly targeted by miR-22-3p (Zhou et al., 2017). MIAT may function as a competing endogenous RNA to positively regulate DAPK2 expression by sponging miR-22-3p, which consequently leads to cardiomyocyte apoptosis involved in the pathogenesis of DCM. In ischemia reperfusion injury, DAPK2 was confirmed as a target of miR-133a. In H9C2 cells, ischemia reperfusion caused a sharp decrease in miR-133a expression and a significant upregulation of DAPK2 expression (Li et al., 2015) (Table 1; Figure 3).

### Non-coding RNAs Involved in the Transcription Regulation of Death-Associated Protein kinase3

Compared to DAPK1 and DAPK2, fewer studies investigated the association between DAPK3 and non-coding RNAs. DAPK3 was reported to be a target of miR-17/20a and played an important role in preventing miR-17/20a depletion-induced genome instability or miR-17/20a overexpression triggered tumor formation (Cai et al., 2015). In ovarian cancer, miR-1307 was up-regulated in the chemoresistant epithelial ovarian cancer tissues and might play a role in the development of chemoresistance in ovarian cancer by targeting DAPK3 (Zhou et al., 2015) (Table 1; Figure 3).

## Bioinformatics Analysis of Potential Non-coding RNAs Involved in the Transcription Regulation of Death-Associated Protein kinases

Although there have been many studies on the non-coding RNAs regulating DAPKs, bioinformatic tools are available to computationally predict new non-coding RNAs for DAPKs, which may help us have a better understanding about the regulation of DAPKs.

### Analysis of Potential miRNAs Involved in the Transcription Regulation of Death-Associated Protein kinases

**FIGURE 4:**
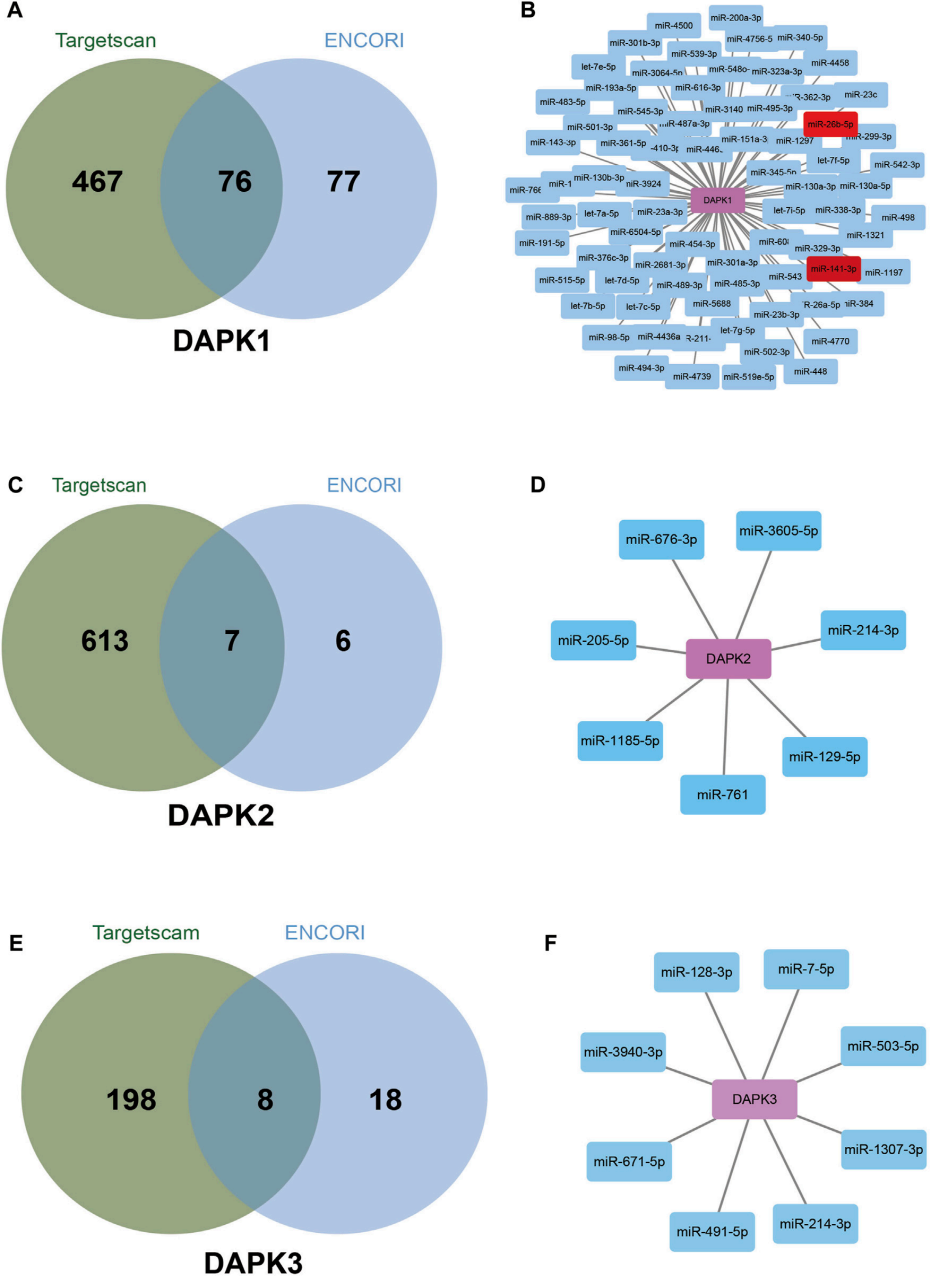
Bioinformatics analysis of potential miRNAs in the regulation of DAPKs. Venn diagrams show potential miRNAs in the regulation of DAPK1 **(A)**, DAPK2 **(C)** and DAPK3 **(E)** based on Targetscan and ENCORI website. Network connection diagram showing potential miRNAs in the regulation of DAPK1 **(B)**, DAPK2 **(D)** and DAPK3 **(F)** based on Targetscan and ENCORI website.

By analyzing a large set of Ago and RBP (RNA binding protein) binding sites derived from all available CLIP-Seq experimental techniques (PAR-CLIP, HITS-CLIP, iCLIP, CLASH), ENCORI (http://starbase.sysu.edu.cn/index.php) have shown extensive and complex RNA–RNA interaction networks (Li et al., 2014; Fan et al., 2020). Targetscan (http://www.targetscan.org/vert_72/) predicts biological targets of miRNAs by searching for the presence of conserved sites that match the seed region of each miRNA (Agarwal et al., 2015). The overlapping target genes in these two databases were considered as miRNA-target genes. According to the prediction of Targetscan and ENCORI databases, 543 miRNAs and 153 miRNAs for DAPK1 were identified respectively. The intersection of the two predictions includes 76 miRNAs such as miR-362-3p, miR-329-3p, miR-141-3p, miR-26b-5p, miR-483-5p and miR-98-5p et al. (Figures 4A,B). Among them, miR-141-3p and miR-26b-5p have been reported to regulate DAPK1 expression (Cui et al., 2018; Su et al., 2019). Similarly, the intersection for DAPK2 and DAPK3 were seven miRNAs and eight miRNAs respectively (Figures 4C–F).

### Analysis of Potential lncRNAs Involved in the Transcription Regulation of Death-Associated Protein kinases

**FIGURE 5:**
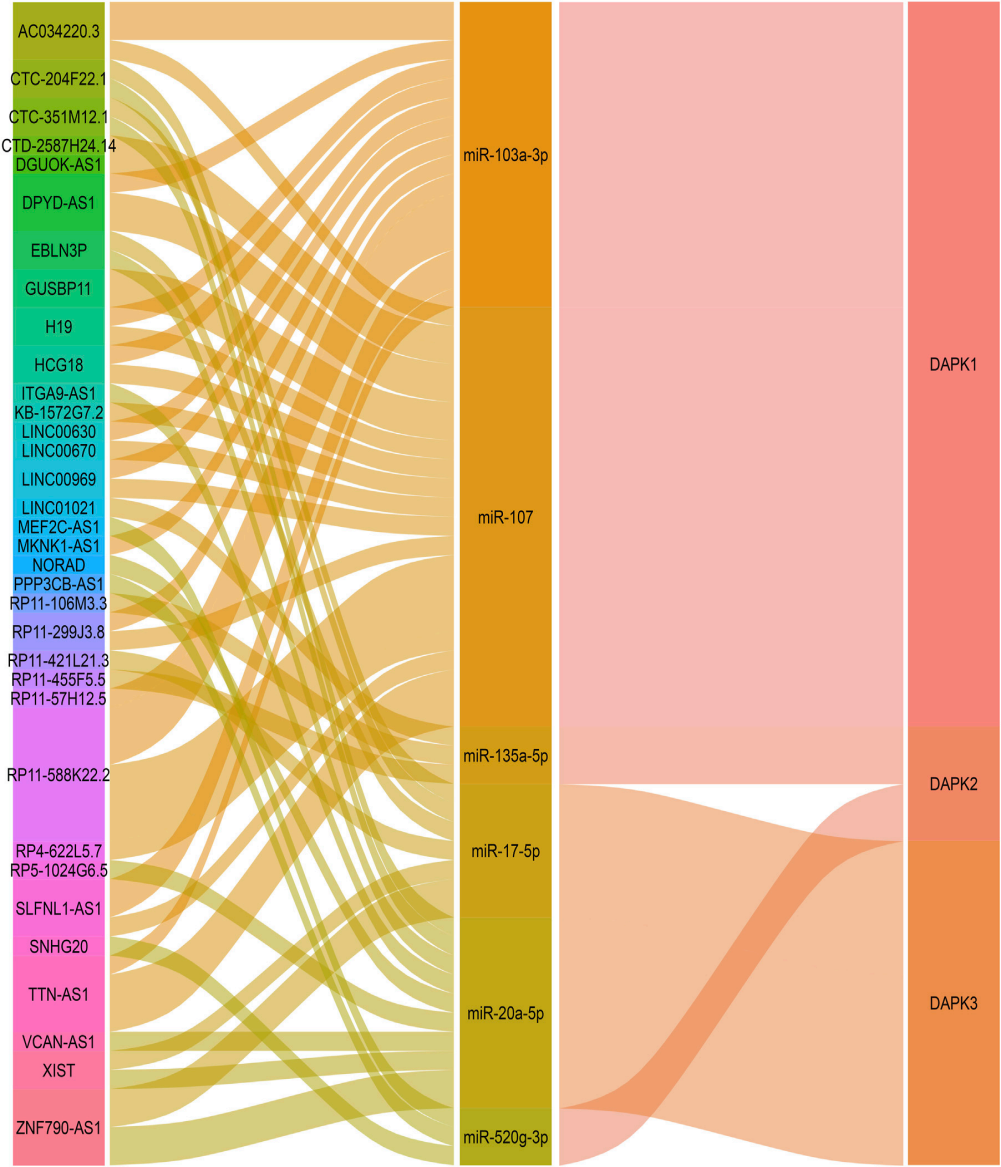
Bioinformatics analysis of potential lncRNAs in the regulation of DAPKs. Sankey diagram for the ceRNA network in DAPK1. Each rectangle represents a gene, and the connection degree of each gene is visualized based on the size of the rectangle.

LncACTdb 2.0 (http://www.bio-bigdata.net/LncACTdb/) is a database which provides comprehensive information of competing endogenous RNAs (ceRNAs) (Wang et al., 2019). We used LncACTdb 2.0 database to discover lncRNAs that may regulate DAPKs *via* ceRNAs mechanism. Base on the bioinformatics analysis, 38 lncRNA-miRNA-DAPK1 mRNA ceRNA network, six lncRNA-miRNA-DAPK2 mRNA ceRNA network and 17 lncRNA-miRNA-DAPK3 mRNA ceRNA network were constructed (Figure 5). 38 lncRNA-miRNA-DAPK1 mRNA ceRNA network has 18 lncRNA including TTN-AS1, RP11-299J3.8, LINC00630, MKNK1-AS1, SLFNL1-AS1, HCG18, RP11-57H12.5, CTD-2587H24.14, DGUOK-AS1, RP11-588K22.2, RP4-622L5.7, H19, GUSBP11, LINC00969, AC034220.3, DPYD-AS1, LINC00670 and KB-1572G7.2. GUSBP11 is significantly higher in gastric cancer plasma compared with healthy controls (Zheng et al., 2019). DGUOK-AS1 promotes the proliferation cervical cancer by sponging miR-653-5p and regulating EMSY (Yan et al., 2020). LINC00630 promotes radio-resistance by regulating BEX1 gene in colorectal cancer cells (Liu et al., 2020). HCG18 contributes to the progression of hepatocellular carcinoma *via* miR-214-3p/CENPM (Zou et al., 2020). TTN-AS1 promotes the progression of lung adenocarcinoma, cholangiocarcinoma and colorectal cancer (Jia et al., 2019; Zhu et al., 2020a). H19 is abnormally expressed in human malignant tumors, and regulates cell proliferation, migration and *via* various mechanisms (Ye et al., 2019). Six lncRNA-miRNA-DAPK2 mRNA ceRNA network has six lncRNA including PPP3CB-AS1, SNHG20, MEF2C-AS1, RP11-455F5.5, RP11-106M3.3 and LINC01021. It has been widely reported that SNHG20 is elevated in various cancers, indicating that SNHG20 may participate in cancer initiation and development (Zhu et al., 2020b). MEF2C-AS1 is identified as a novel biomarker in diffuse gastric cancer (Luo et al., 2018). 17 lncRNA-miRNA-DAPK3 mRNA ceRNA network has 10 lncRNA including ZNF790-AS1, NORAD, EBLN3P, XIST, RP5-1024G6.5, CTC-351M12.1, CTC-204F22.1, ITGA9-AS1, RP11-421L21.3 and VCAN-AS1. EBLN3P promotes the progression of liver cancer by regulating miR-144-3p/DOCK4 (Li et al., 2020). VCAN-AS1 contributes to the progression of gastric cancer *via* regulating p53 expression (Feng et al., 2020). NORAD is a highly conserved lncRNA necessary for genome stability and is dysregulated in various cancers (Soghli et al., 2021). XIST is associated with poor prognosis and metastasis of cancer in patients (Yin et al., 2019).

### Analysis of Potential circRNA Involved in the Transcription Regulation of Death-Associated Protein kinases

**FIGURE 6:**
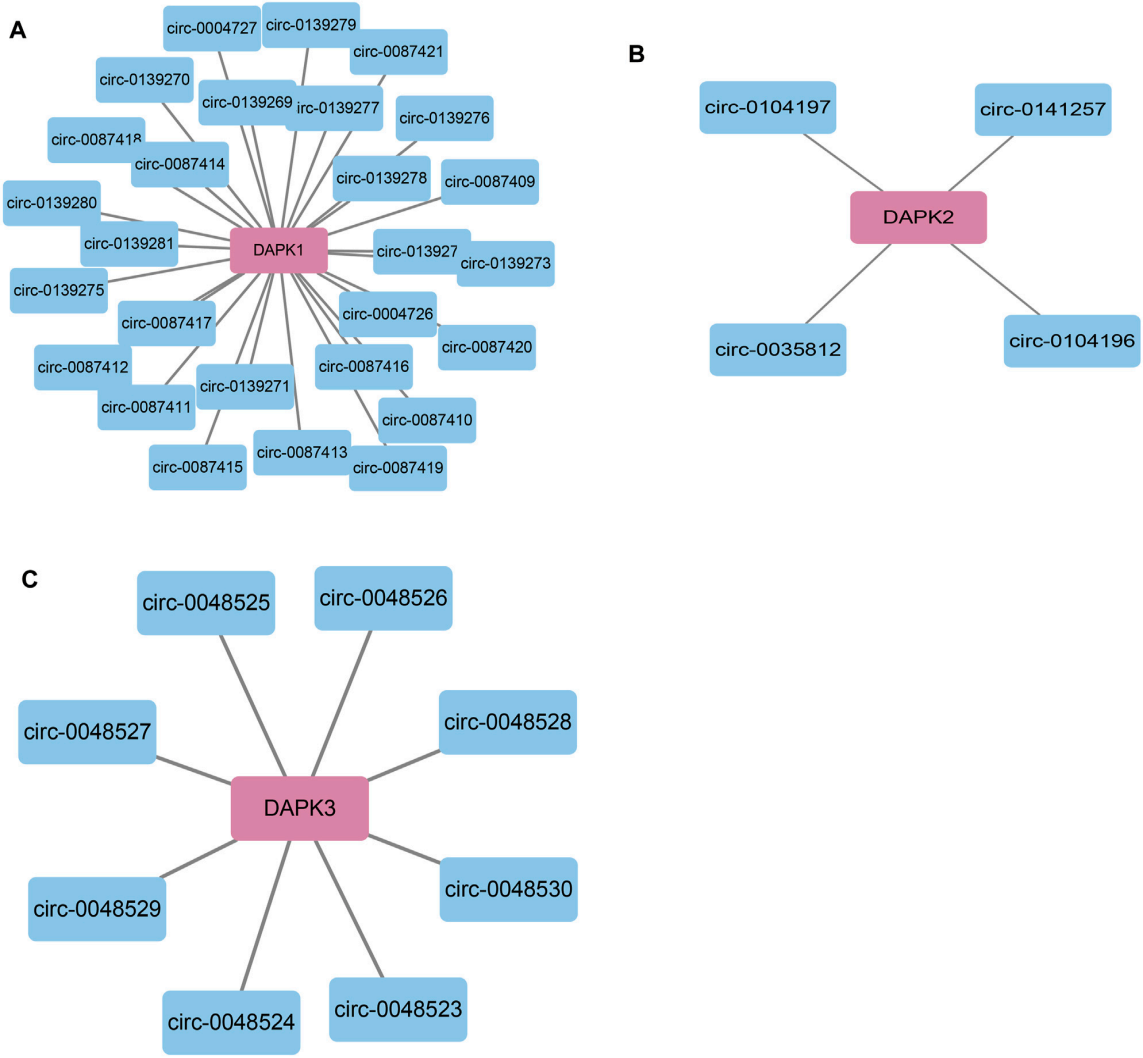
Bioinformatics analysis of potential circRNAs in the regulation of DAPKs. Bioinformatics analysis of potential circRNA in the regulation of DAPK1 **(A)**, DAPK2 **(B)** and DAPK3 **(C)**.

CircBase (http://www.circbase.org/) is a database for circular RNAs (Glažar et al., 2014). Using CircBase, we found 27 circRNAs that may regulate DAPK1 including circ-0087415, circ-0139281 and circ-008742 et al., four circRNAs for DAPK2 including circ-0104197, circ-0141257, circ-0104196 and circ-0035812 and eight circRNAs for DAPK3 including circ-0048524, circ-0048528, circ-0048527, circ-0048525, circ-0048526, circ-0048530, circ-0048523 and circ-0048529 (Figure 6).

## Conclusion

In the review, we conducted a comprehensive review of studies on non-coding RNAs and DAPKs to understand the current research status, and to explore the potential regulatory miRNAs, lncRNAs and circRNAs for the expression of DAPKs. Based on the review of current evidence, we have presented the regulatory network linking miRNAs, lncRNAs and DAPKs. It should be noted that much of our current understanding of non-coding RNAs in the transcription regulation of DAPKs comes from studies of a small number of non-coding RNAs and it is presently unclear whether these are representative of the group as a whole. Any review on this subject of non-coding RNAs in the regulation of DAPKs will always be incomplete for the reason that the field is still expanding. In addition to the miRNAs and lncRNAs described here, the circRNAs participate in the regulation of DAPKs remains an unexplored area of investigation. One thing that seems likely is that as we begin to identify more non-coding RNAs regulating DAPKs, the regulatory mechanisms of DAPKs in cancers and disease will be better understood.

It is well known that one non-coding RNA regulates multiple genes, and one gene may be regulated by multiple non-coding RNAs. As demonstrated in this review, the expression of DAPK1 is regulated by multiple non-coding RNAs. One important line for future research will be to identify the key non-coding RNAs participating in the transcription regulation of DAPKs in specific types of cancer.

Bioinformatics analyses have in the past displayed a strong ability to predict and construct the coding gene and non-coding gene co-expression network. Previous study has in the past tended to focus only on the miRNAs in the transcription regulation of DAPKs. For example, DAPK1 has been confirmed to be regulated by miR-191, miR-483-5p, miR-141-3p, miR-98, miR-141-3p and miR-26a-5p. In prediction websites ENCORI and Targetscan, these miRNAs were predicted to bind to DAPK1 3′ UTR. MiR-129-5p, miR-205-5p, miR-214-3p, miR-1185-5p, miR-761, miR-3605-5p and miR-676-3p were found to be involved in the transcription regulation of DAPK2, these miRNAs were predicted to bind to DAPK2 3′ UTR in Targetscan websites. Compared to miRNAs, there has been little research on lncRNAs and circRNAs targeting DAPKs, and only three lncRNAs were reported to be involved in the transcription regulation of DAPKs. As it becomes clear that lncRNA and circRNA have a distinct biological role, it is logical to discovered that lncRNA and circRNA that regulate DAPKs will be found to have clinical implications and also be worthy of investigation. Base on LncACTdb website, we found that a total of two miRNAs, miR-103a-3p and miR-107, were involved in the 38 lncRNA-miRNA-DAPK1 ceRNA network. Meanwhile, miR-103a-3p and miR-107 were also predicted to bind to DAPK1 3’ UTR through ENCORI website. These results imply that miR-103a-3p and miR-107 may play an important role in the transcription regulation of DAPK1. Of additional concern, lncRNA DAPK1-IT1 is transcribed from an intron of the DAPK1 gene. DAPK1-IT1 has been reported to regulate cholesterol metabolism and inflammatory response in macrophages and promotes atherogenesis by sponging miR-590-3p and regulating LPL (lipoprotein lipase), and possibly linked to respiratory diseases. Thus, the interaction between DAPKs and the ncRNAs can be complex (Zhen et al., 2019; Liao et al., 2020).

In conclusion, this review integrates and predicts the potential non-coding RNAs that may participate in the transcription regulation of DAPKs. We hope this could help speed up the research on non-coding RNAs in the transcription regulation of DAPKs in the future.
